# Lupus and recurrent pregnancy loss: the role of female sex hormones and B cells

**DOI:** 10.3389/fendo.2023.1233883

**Published:** 2023-10-03

**Authors:** Natalin Jimena Valeff, Maria Silvia Ventimiglia, Lianghui Diao, Federico Jensen

**Affiliations:** ^1^ Center for Pharmacological and Botanical Studies (CEFYBO-UBA-CONICET), Medical Faculty, Buenos Aires University, Buenos Aires, Argentina; ^2^ Shenzhen Key Laboratory of Reproductive Immunology for Peri-implantation, Shenzhen Zhongshan Institute for Reproduction and Genetics, Fertility Center, Shenzhen Zhongshan Urology Hospital, Shenzhen, China; ^3^ Centro Integrativo de Biología Y Química Aplicada. Universidad Bernardo O’Higgins, Santiago, Chile

**Keywords:** recurrent pregnancy loss, lupus, B cells, hormones, pregnancy

## Abstract

Systemic lupus erythematosus is a debilitating autoimmune disease characterized by uncontrolled activation of adaptive immunity, particularly B cells, which predominantly affects women in a 9 to 1 ratio compared to men. This stark sex disparity strongly suggests a role for female sex hormones in the disease’s onset and progression. Indeed, it is widely recognized that estradiol not only enhances the survival of autoreactive B cells but also stimulates the production of autoantibodies associated with systemic lupus erythematosus, such as anti-nuclear antibodies and anti-dsDNA antibodies. Clinical manifestations of systemic lupus erythematosus typically emerge after puberty and persist throughout reproductive life. Furthermore, symptoms often exacerbate during the premenstrual period and pregnancy, as increased levels of estradiol can contribute to disease flares. Despite being fertile, women with lupus face a heightened risk of pregnancy-related complications, including pregnancy loss and stillbirth, which significantly surpass the rates observed in the healthy population. Therefore, this review aims to summarize and discuss the existing literature on the influence of female sex hormones on B-cell activation in patients with systemic lupus erythematosus, with a particular emphasis on their impact on pregnancy loss.

## 1 Introduction

Recurrent pregnancy loss (RPL) is a distressing pregnancy disorder experienced by ~2.5% of women trying to conceive. It is defined as the spontaneous demise of two or more clinically recognized pregnancies before the fetus reaches viability; RPL includes embryonic and fetal losses from the time of conception until 24 weeks of gestation (1, 2).

Autoimmune disorders have been included along with chromosomal errors, anatomical uterine defects, and endometrial dysfunction as the most common etiologies linked to RPL (3). Indeed, certain features commonly associated with autoimmune diseases, such as inappropriate complement activation (4–6) and the prevalence of specific autoantibodies (4, 7–11) show strong associations with RPL.

Furthermore, systemic autoimmune diseases, including systemic lupus erythematosus (SLE), have been identified as significant risk factors for RPL, similar to other autoimmune conditions (12).

SLE is a chronic autoimmune disease that predominantly affects women of reproductive age compared to men and has the potential to affect any organ in the body (13–15). The intricate clinical presentation and pathogenesis of SLE make its definition exceptionally challenging. According to the European League Against Rheumatism (EULAR) and the American College of Rheumatology (ACR), the classification criteria for SLE consist of a mandatory entry criterion of positive anti-nuclear antibodies (ANAs) at least once, followed by additive weighted criteria grouped into seven clinical domains, namely, constitutional, hematologic, neuropsychiatric, mucocutaneous, serosal, musculoskeletal, and renal, and three immunological domains: antiphospholipid antibodies (aPLs), complement proteins, and SLE-specific antibodies (16). ANAs are a group of autoantibodies that target components of the cell nucleus and can bind to proteins, nucleic acids, and protein–nucleic acid complexes (17).

From an immunological perspective, the intricate interplay of environmental, genetic, and hormonal factors results in dysregulation and abnormal activation of the innate and adaptive immune system. This leads to the generation of pathogenic autoantibodies, such as ANAs, anti-double-stranded DNA antibodies (anti-dsDNA), and aPLs, as well as the deposition of immune complexes, ultimately causing tissue damage (18, 19).

Moreover, the impact of ANAs (20) and the presence of various types of aPLs (21) significantly varies between women with RPL and autoimmune diseases, in comparison to those without autoimmunity (22). Indeed, the rate of pregnancy loss among patients with SLE is substantially higher compared to the general healthy population (3). Furthermore, the stage of SLE that the patient is in at the moment of becoming pregnant, including disease activity and renal involvement, not only impacts the health status of the mother but may also influence fetal and neonatal outcomes (23, 24). In this regard, several studies have found that increased serum levels of IL-6, IL-10, and INF-α in patients with SLE are associated with disease activity (25, 26). Regarding disease activity at the time of conception, numerous prospective studies have recently shown that women with inactive SLE generally experience minimal flares during pregnancy, while those with active SLE face an elevated risk of adverse maternal and fetal outcomes (27–29). These findings are consistent with previous reports, indicating that the rate of live births is lower in patients with clinically active SLE in the 6 months before conception compared to those with inactive disease prior to conception (30). Furthermore, RPL among women with SLE appears to be linked to a higher rate of fetal death, which is associated with the presence of aPLs (31, 32). Furthermore, it is well established that newborn babies born to mothers with SLE can develop neonatal lupus, a rare condition that is not a form of SLE, but rather a condition that affects the newborn due to the transfer of maternal autoantibodies across the placenta during pregnancy (33).

Considering the sex and age predisposition of SLE, female sex hormones are undeniably involved in the pathogenesis of the disease (34). Studies conducted on SLE-prone mice using gonadectomy/hormone deprivation and hormone supplementation have consistently confirmed this association, revealing that estrogen exacerbates the disease, while its removal ameliorates the disease in female subjects [reviewed in (35)]. In the context of pregnancy, the increase in female sex hormone levels may influence or potentiate the abnormal function of the immune cells in patients with SLE, thereby exacerbating the disease symptoms and leading to pregnancy complications, including RPL (36, 37).

Considering all the evidence, the objective of this review is to examine the current state of knowledge regarding the impact of preexisting SLE on the development of RPL, with particular focus on the role of female sex hormones in B cell activation and autoantibody production.

## 2 Pregnancy in patients with systemic lupus erythematosus: the impact on recurrent pregnancy loss

As mentioned earlier, SLE predominantly affects women during their reproductive age, when individuals may seek to become pregnant. However, while fertility is generally preserved in women with SLE, pregnancy in these patients can be associated with adverse maternal and fetal outcomes, including RPL (38). In a recent meta-analysis of pregnancy studies published from 2017 to 2019, it was shown that patients with SLE had markedly increased risk of stillbirth (risk ratio (RR) 16.49, 95% CI 2.95 to 92.13; p =0 .001) and fetal loss (RR 7.55, 95% CI 4.75 to 11.99; p = 0.00001) compared to healthy pregnant women (39). Despite substantial declines in rates of pregnancy loss among patients with SLE in recent years, they remain higher compared to the healthy population (40). Indeed, approximately 20% of pregnancies in patients with SLE result in miscarriages (40).

Several biomarkers have been investigated as potential predictors of pregnancy complications in women with SLE. Notably, aPLs, including anticardiolipin antibodies and lupus anticoagulants, have been associated with obstetric complications such as RPL, recurrent implantation failure, pre-eclampsia, and preterm birth (41, 42). Additionally, research has shown that low levels of complement proteins, such as C3 and C4 during the first trimester are associated with an increased risk of pregnancy loss (43) in patients with SLE.

Although the causes behind poor pregnancy outcomes in patients with SLE are diverse, there is a general consensus that active disease, characterized by the activation of autoreactive B cells and production of autoantibodies, at the time of conception and during pregnancy significantly impacts maternal and fetal outcomes (38). This is not surprising, given that a successful pregnancy relies on a precisely regulated balance between maternal immune activation and immune tolerance (44). Any disruptions or imbalances in this delicate equilibrium can lead to pregnancy loss. Conversely, during pregnancy, an increase in the levels of female sex hormones can promote B cell autoreactivity and exacerbate the symptoms of SLE, creating a negative feedback loop. This phenomenon leads to the activation of various immune mechanisms, which can not only worsen the symptoms of SLE but also contribute to pregnancy loss. Therefore, the hormonal regulation of B cell activation during SLE and its implication in pregnancy loss will be discussed in greater detail below.

## 3 The impact of female sex hormones on B cell activation in patients with systemic lupus erythematosus

B cells are essential components of the adaptive immune system responsible for antibody production. They can be classified into marginal zone (MZ), B1, and B2 B cells based on their phenotype, localization, and functionality (45). While T cell activation relies on antigen presentation by antigen-presenting cells (APCs), B cells, on the other hand, can directly interact with antigens through their receptor (B cell receptor, BCR) (46). However, apart from the signal provided by antigens through BCR, B cells require a second signal for proper activation, which can be delivered by toll-like receptors (TLRs), BAFF-R, or BCR cross-linking in the case of MZ and B1 B cells (47). On the other hand, upon antigen recognition, B2 B cells migrate to the germinal center, where they receive a second signal from follicular T-helper (Tfh) cells. Subsequently, they mature into either antibody-producing plasma cells or memory B cells.

Female sex hormones play a significant role in the development and activity of the immune system (48). Both innate and adaptive immune cells bear receptors for sex hormones and respond to hormonal cues (49). Women display higher frequencies of B cells (50) along with enhanced B cell survival, maturation, and class switching. They also demonstrate greater antibody responses and higher basal levels of immunoglobulins (Igs) compared to men (51), suggesting the involvement of female sex hormones in controlling diverse B cell functions. Indeed, estrogen has been shown to reduce the production of B cell precursors, impair B cell tolerance, and increase the activation and survival of autoreactive B cells (52, 53). While B cells express both estrogen receptor (ER) α and β, it is ERα that predominantly regulates BCR signal strength (54). Elevated levels of estrogen and ERα engagement result in reduced BCR signal strength and modulation of survival regulators such as Bcl-2, CD22, and SH2-containing protein tyrosine phosphatase (SHP)-2, thereby suppressing apoptosis (52). Moreover, elevated estrogen levels result in increased serum BAFF levels, which, together with reduced BCR signal strength, promote the survival of autoreactive B cells that would otherwise be eliminated from the naive repertoire. Consequently, these autoreactive B cells gain entry into the mature B cell pool (55, 56). In such circumstances, heightened estrogen stimulation on B cells triggers a breakdown of tolerance and uncontrolled proliferation and enhances the survival of high-affinity DNA-reactive B cells, which may potentially lead to autoimmunity (54).

A significant proportion of autoreactive B cells originates from the B2 B cell pool, which requires second signals provided by follicular T helper cells to complete their activation. The significance of these pathways in promoting autoantibody production has been demonstrated in genetically modified lupus-prone mice and using blocking antibodies against various costimulatory molecules, such as inducible costimulatory ligand (ICOS-L) and CD40 ligand. Consequently, T helper cells play a crucial role in the development and progression of SLE disease (57). Furthermore, Tfh cells not only express estrogen receptors, but it has also been demonstrated that estradiol promotes the expansion of Tfh cells and, consequently, enhances the humoral immune response (58). Therefore, in the context of SLE, estradiol appears to exert its effects on the Tfh/B2 B cell axis, promoting the development and survival of autoreactive B cells.

The fact that 90% of patients with SLE are women clearly highlights a strong sex bias in this autoimmune disease. Several hypotheses have been proposed to explain this phenomenon, with the influence of female sex hormones being the most widely accepted (59). In this regard, it is known that the clinical manifestation of the disease typically appears after puberty, affecting women between the ages of 20 to 50, a period during which levels of estradiol and progesterone significantly rise (59). The strongest evidence supporting the role of female sex hormones in SLE comes from the observation that patients with SLE experience disease exacerbation during the premenstrual period and in pregnancy (35, 59). Interestingly, a case report demonstrated that administering cross-gender hormones to a transgender female resulted in lupus nephritis, and the withdrawal of estradiol supplementation upon admission prevented the worsening of symptoms. This provides further support for the role of estradiol in driving SLE (60). Animal studies also provide support for the role of estrogen in SLE. Ovariectomized lupus-prone mice showed ameliorated disease, while estrogen supplementation in castrated male mice worsened the symptoms [reviewed in (35)]. Moreover, targeted deletion of ERα specifically in B cells has been shown to reduce the production of pathogenic autoantibodies and the development of nephritis in lupus-prone mice (61). Additionally, tamoxifen treatment significantly reduced autoantibody production and improved the course of SLE in SLE-prone mice (62).

In pregnant SLE patients, estrogen levels and ERα expression not only mediate the increase in anti-dsDNA but also alter the B-cell repertoire, leading to the expansion of autoreactive clones (63, 64). As a result, hormone levels during pregnancy have a substantial impact on the function of autoreactive B cells, intensifying SLE symptoms and contributing to adverse pregnancy outcomes, including RPL (36, 37). In fact, E2 has been demonstrated to decrease B-cell lymphopoiesis in the bone marrow at the pro–B-cell stages in mice and to alter transitional 2 (T2) B cell maturation, both during pregnancy and in patients with SLE (53, 65). Under SLE conditions, elevated BAFF levels and reduced BCR signal strength can lead to the maturation of transitional B cells into a marginal zone (MZ) B cell expansion. Under specific conditions, marginal zone (MZ) B cells can serve as precursors of unswitched memory B cells without T cell help (66). It has been previously demonstrated that during pregnancy, there is a bias toward the development of marginal zone (MZ) B cells (67). This, along with the abnormal differentiation of unswitched memory B cells observed in patients with SLE (68) may pose a risk to the successful development of pregnancy in patients with SLE. In fact, an increase in unswitched memory B cells is observed in patients with a history of RPL and obstetric complications (69, 70).

Therefore, the presence of autoreactive B cells, along with increased B cell activation and autoantibody production in patients with SLE, poses significant challenges when it comes to achieving a full-term pregnancy.

## 4 B-cell activation and autoantibody production in lupus: impact on pregnancy well-being

Upon activation, B cells undergo a series of tightly regulated processes that culminate in the differentiation of highly specialized cells capable of producing antibodies, as well as memory B cells (45). In addition to antibodies, activated B cells can produce a wide range of cytokines, especially when their activation goes through their BCR together with BAFF-R (71). Signaling through the BAFF-R activates several downstream pathways, including NF-KB, ERK, and MAPK, which regulate the survival functions of immature, transitional, and mature B cells (72, 73). Interestingly, it has been demonstrated that B cells from pregnant women show downregulation of transcripts associated with these pathways (74) along with reduced levels of BAFF in serum as pregnancy progresses (75), suggesting that B cells are less susceptible to being activated during pregnancy. Indeed, a transcriptomic analysis performed on B cells isolated from pregnant mice confirmed that several B cell activation pathways, including BCR, TLR, and BAFF-R, are significantly diminished compared to B cells from non-pregnant control animals (44). Furthermore, a study by Valeff et al. (44) found that B cells isolated from pregnant women in the first trimester of pregnancy produced significantly lower levels of inflammatory cytokines when activated through their BCR and TLRs compared to B cells from non-pregnant women, reinforcing the notion of B cells being less susceptible to activation, at least during the early stages of pregnancy.

In the context of SLE, aberrant B-cell activation plays a significant role in the pathogenesis of the disease. Dysregulation of BCR and BAFF-R pathways are common and dominant factors involved in this aberrant B-cell activation (76). Furthermore, patients with SLE exhibit elevated levels of BAFF in their serum (77–79), strongly indicating the involvement of the BAFF-R pathway in B cells as a key component of SLE pathology. Indeed, mice overexpressing BAFF develop a lupus-like disease characterized by the production of ANAs and anti-dsDNA (80).

In the context of pregnancy, while the production of natural and protective antibodies is related to pregnancy success (81, 82), the presence of autoantibodies is associated with RPL (8). There is growing evidence suggesting that ANAs can play a role in both early pregnancy complications, such as embryo implantation, and pregnancy loss (83). While low titers of ANAs are common in healthy women, those with RPL often exhibit high titers of ANAs (>1:160) (83). Moreover, ANAs have been suggested to have a direct effect on the quality and development of oocytes and embryos, leading to reduced implantation rates (84). In the fetal-maternal interface, ANAs can induce the precipitation of immune complexes, attributed to elevated C3 levels, resulting in T cell activation and increased production of inflammatory cytokine (IFN-α), which in turn stimulates the humoral immune response (85, 86). Complement activation rapidly increases the production of the pro-inflammatory cytokine TNF, which in turn recruits inflammatory cells into the placenta, ultimately contributing to pregnancy loss (87).

It is well known that imbalances toward a pro-inflammatory milieu are associated with poor pregnancy outcomes (88). Moreover, the TNF/IL-10 ratio in serum is used as an indicator or predictor of pregnancy loss (89). In line with this, the production of IL-10 by B cells is considered essential for successful pregnancies (90). Interestingly, in patients with SLE, there is a significant decrease in IL-10 production by B cells (91). Even though, the elevated serum levels of IL-10 observed in pregnant women with SLE compared to controls (25) would be an advantage in normal pregnancy conditions, the immunosuppressive and anti-inflammatory effects of this cytokine are impaired in patients with SLE compared to healthy individuals (92).

Therefore, it is reasonable to speculate that uncontrolled B cell activation in patients with SLE during gestation may lead to the production of pro-inflammatory cytokines and harmful antibodies, which could potentially compromise the well-being of the pregnancy.

**Figure 1:**
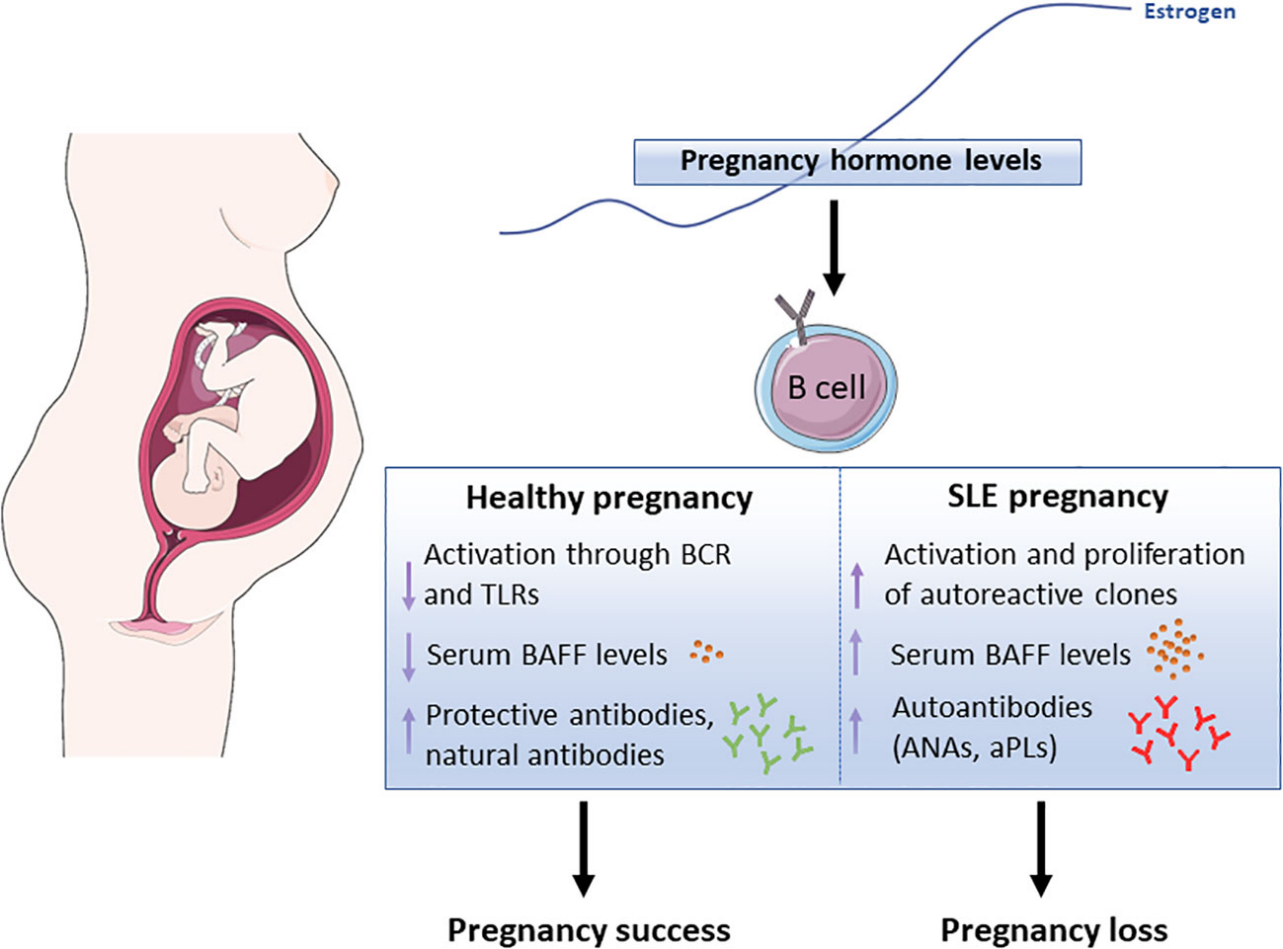
Schematic representation showing the potential effect of pregnancy-associated hormones, in particular estrogen, in B cell functions both, in healthy pregnancy and lupus pregnancy. BCR (B cell receptor), TLR (Toll-Like Receptor), BAFF (B Cell-Activating Factor), ANAs (anti-Nuclear Antibodies), aPLs (anti-Phospholipid Antibodies). The Figure was partly generated using Servier Medical Art, provided by Servier, licensed under a Creative Commons Attribution 3.0 Unported License (https://creativecommons.org/licenses/by/3.0/)

In conclusion, maintaining a balanced B-cell activation is essential for a successful pregnancy. In women with preexisting SLE, hormonal changes may disrupt this balance, leading to the production of inflammatory cytokines and autoantibodies. This dysregulation can exacerbate disease symptoms and contribute to pregnancy complications, including RPL. Therefore, understanding the impact of B-cell activation and its relationship with hormonal changes during gestation is crucial for managing SLE and optimizing pregnancy outcomes (**Figure 1**).

## Author contributions

NV designed, drafted, and revised the work. MV designed and drafted the work. LD drafted and revised the work. FJ designed, drafted, supervised, and revised the work. All authors contributed to the article and approved the submitted version.
